# The Evaluation of Blood Prooxidant–Antioxidant Balance Indicators and Cortisol Pre- and Post-Surgery in Patients with Benign Parotid Gland Tumors: A Preliminary Study

**DOI:** 10.3390/jcm14155425

**Published:** 2025-08-01

**Authors:** Sebastian Bańkowski, Jan Pilch, Bartosz Witek, Jarosław Markowski, Wirginia Likus, Michał Rozpara, Ewa Sadowska-Krępa

**Affiliations:** 1 Department of Biochemistry, Institute of Sport Sciences, Academy of Physical Education, 40-065 Katowice, Poland; s.bankowski@awf.katowice.pl; 2 Department of Anatomy and Pre-Medical First Aid, Institute of Sport Sciences, Academy of Physical Education, 40-065 Katowice, Poland; j.pilch@awf.katowice.pl; 3 Department of Laryngology, Faculty of Medical Sciences in Katowice, Medical University of Silesia, 40-959 Katowice, Poland; witek.b95@gmail.com (B.W.); jmarkowski@sum.edu.pl (J.M.); 4 Department of Anatomy, Faculty of Health Sciences in Katowice, Medical University of Silesia, 40-959 Katowice, Poland; wlikus@sum.edu.pl; 5 Department of Health Training, Institute of Sport Sciences, Academy of Physical Education, 40-065 Katowice, Poland; m.rozpara@awf.katowice.pl

**Keywords:** oxidative stress, parotid gland tumors, surgery

## Abstract

**Background:** The majority of parotid gland tumors are benign, e.g., pleomorphic adenoma (PA) and Warthin’s tumor (WT). From a biomedical point of view, oxidative stress is of significant importance due to its established association with the initiation and progression of various types of cancer, including parotid gland cancers. This study aimed to assess whether blood prooxidant–antioxidant markers could aid in diagnosing and guiding surgery for recurrent malignancies after parotid tumor treatment. **Methods:** We examined patients (n = 20) diagnosed with WT (n = 14) and PA (n = 6) using histopathological verification and computed tomography (CT) who qualified for surgical treatment. Blood samples were taken before the surgery and again 10 days later for biochemical analysis. The activities of the antioxidant enzymes (SOD, CAT and GPx), the non-enzymatic antioxidants (GSH and UA) and oxidative stress markers (MDA and TOS) were determined in the blood. The activities of CK and LDH and the concentrations of Cor and TAS were measured in the serum. Hb and Ht were determined in whole blood. **Results:** The patients’ SOD, CAT, and GPx activities after surgery did not differ significantly from their preoperative levels. However, following surgery, their serum TOS levels were significantly elevated in all the patients compared to baseline. In contrast, the plasma MDA concentrations were markedly reduced after surgery. Similarly, the GSH concentrations showed a significant decrease postoperatively. No significant changes were observed in the CK and LDH activities, TAS concentrations, or levels of Hb, Ht and Cor following surgery. **Conclusions:** The surgical removal of salivary gland tumors did not result in a reduction in oxidative stress at 10 days after surgery. Therefore, further studies are needed to determine the effectiveness of endogenous defense mechanisms in counteracting the oxidative stress induced by salivary gland tumors.

## 1. Introduction

Tumors of the salivary glands, which develop in the head and neck, account for about 3–6% of tumors. Approximately 70–85% of these tumors occur in the parotid glands, of which 10–17% are malignant and the majority are benign [1,2]. Recently, there has been a significant increase in the incidence of salivary gland parotid cancers [1]. It is believed that the main risk factors for parotid gland tumorigenesis are long-term smoking, ionizing and electromagnetic radiation, and viral infections (including HPV) [1]. Most parotid gland tumors are benign, and the two most common are pleomorphic adenoma (PA, accounting for 60% of all benign salivary gland tumors), also known as mixed tumor, and Warthin’s tumor (WT, accounting for 5–30% of all salivary gland tumors), known as cystadenoma lymphomatosum [3,4].

Correct diagnosis of tumors is essential to ensure appropriate treatment and follow-up [5]. The list of biomarkers used to diagnose these lesions is extensive and includes, but is not limited to, Cytokeratin 7 (CK7) [5], the polycystic adenoma-1 (PLAG1) gene [6,7], mammaglobin [8], the anoctamin-1 gene (also known as DOG-1) [9], HMG-box 10-related SRY (SOX10) [10,11], gross cystic disease fluid protein-15 (GCDFP-15) [5], human epidermal growth factor receptor-2 (HER-2) [12], c-kit protooncogene (CD117) [13], GATA-binding protein 3 (GATA3) [11], nuclear receptor subfamily 4 group A member 3 (NR4A3) [14], and insulinoma-related transcription factor protein 1 (INSM1) [15]. On the other hand, prognostic biomarkers whose presence or value correlates with disease course and patient prognosis include Ki67 [16,17], minichromosome maintenance 2 (Mcm-2) [16], programmed death ligand 1 (PD-L1) [18], human epidermal growth factor receptor 2 (HER2) [19], androgen receptor (AR) [19], and mucin markers (e.g., MUC1, MUC4, MUC5AC and MUC5B) [20].

Treatment of these cancers requires surgery, which can also cause surgical complications. The most common postoperative complications of salivary gland tumors are both short-term and long-term. The former include facial nerve palsy, hemorrhage or hematoma, infection, pain, jawbone, sensory deficit in the auricle, and salivary fistula. On the other hand, the latter ones include Frey syndrome, facial collapse after facial nerve palsy, tumor recurrence, soft tissue deficit, and hypertrophic scarring, resulting in decreased quality of life [21,22,23].

From a biomedical point of view, oxidative stress is significantly important because of its association with a number of diseases, i.e., neurodegenerative diseases, cardiovascular diseases, immune system dysfunctions, and cancers [24]. Oxidative stress has been implicated in the origin and development of various cancers including parotid cancers [25]. A balance between the production and elimination of reactive oxygen species (ROS) is necessary to maintain an adequate state of prooxidant–antioxidant balance in cells [26]. ROS are products generated by metabolic reactions in the mitochondria of eukaryotic cells. In normal functioning cells, low concentrations of ROS are required for signal transduction prior to their elimination, while cancer cells, due to their accelerated metabolism, require high concentrations of them to maintain a high rate of cell proliferation [24,27].

In the case of parotid gland tumors (as with all types of cancer), there is a lack of information regarding their pathomechanisms and the use of specific biomarkers to distinguish between benign and malignant tumors [2].

Given the above, the present study was designed to establish whether selected indicators of the blood prooxidant–antioxidant balance could contribute any new information useful for diagnosis and determining whether patients qualify for surgical treatment addressing the recurrence of the “malignant process” after an initial surgery to remove parotid tumors. We tested the hypothesis that surgery would enhance the activities of blood antioxidant enzymes and reduce oxidative stress.

## 2. Materials and Methods

### 2.1. Participants

The sample size was calculated using G*Power version 3.1.9.7, developed by Heinrich Heine University in Düsseldorf, Germany. An effect size of 0.5 was assumed, with an alpha error probability of 0.05 and a test power of 0.84. Based on these indices, the required sample size was determined to be thirty.

The sample consisted of patients diagnosed with a benign tumor of the parotid glands (pleomorphic adenoma (PA), also known as a mixed tumor, or Warthin’s tumor (WT) after histopathological verification and computed tomography (CT)), who were qualified for surgical treatment at the Department of Laryngology of the Silesian Medical University in Katowice. Thirty eligible patients volunteered for this study. Three patients did not qualify for surgery. Biochemical analyses were conducted on twenty individuals (WT: n = 14; PA: n = 6) both prior to and following surgery. The basic anthropometric characteristics of the patients (n = 20) are presented in Table 1.

**Figure 1:**
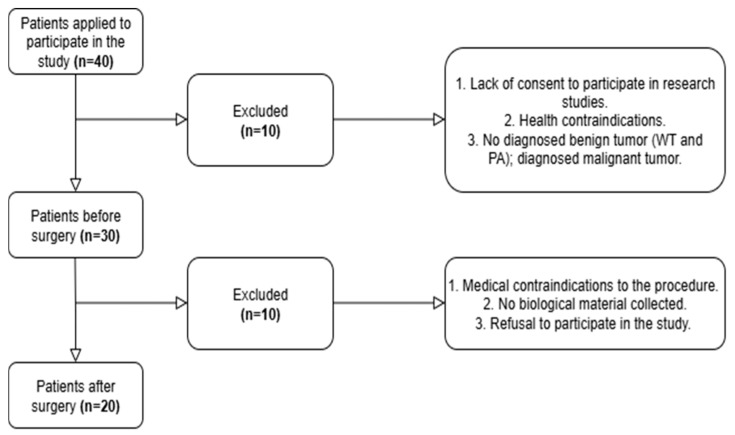
Participant flow chart.

Participants were excluded from the study if they did not provide consent, were unwell on the scheduled surgery date, were deemed ineligible for surgery or had medical contraindications to the procedure, or if biological material could not be collected (Figure 1). To be included, individuals had to be at least 18 years old, provide voluntary informed consent, and have a histopathologically confirmed benign parotid gland tumor diagnosed through computed tomography (CT).

All the patients were informed of the study’s aim and nature and provided their written informed consent. The study protocol conformed to the ethical guidelines of the World Medical Association Declaration of Helsinki and was approved by the Institutional Ethics Committee. Additionally, the study was prospectively registered in the Australian New Zealand Clinical Trials Registry (ANZCTR) under the number ACTRN12624001482550. Recruitment and follow-up occurred between 28 January 2024 and 17 September 2024. The authors confirm that all trials that are ongoing and related to this intervention are registered.

### 2.2. Surgery and Blood Collection

The patients underwent superficial parotidectomy, involving the complete excision of the superficial lobe of the parotid gland while preserving the facial nerve. The computed tomography (CT) scans and fine-needle aspiration (FNA) procedures were performed prior to hospital admission, as part of the patients’ diagnostic work-up in outpatient settings. However, from the moment of hospital admission, the study was conducted in a prospective manner. Patient eligibility for inclusion was confirmed at the time of admission, and all subsequent data, including surgical intervention and final histopathological evaluation, were collected prospectively according to a predefined study protocol.

Blood venous samples for biochemical analysis were obtained prior to surgery and again 10 days postoperatively.

#### 2.2.1. Surgery Methodology

A bayonet skin incision was made in the preauricular region with a length of about 5–8 cm extending from the zygomatic arch around the auricular lobe, over the mastoid process, and then downward behind the angle of the mandible. The soft tissues were stripped with both sharp and blunt instruments. The parotid gland pouch was visualized and opened.

While approaching through the superficial lobe of the parotid gland, enucleation was performed in the case of Warthin’s tumor; however, for pleomorphic adenomas, the superficial lobe of the parotid gland including the tumor was removed. In some cases, ligation of the retromandibular vein was performed, and the great auricular nerve was visualized. The trunk of the facial nerve was identified. Subsequently, the parotid duct was tied off and severed. The superficial lobe of the parotid gland, along with the tumor, was removed in its entirety. The wound was closed in layers over the suction drainage.

#### 2.2.2. Biochemical Analyses

A portion of the fresh whole blood samples was immediately assayed for reduced glutathione (GSH) using a colorimetric method and 5.5′-dithiobis-2-nitrobenzoic acid [28], and hematocrit was assayed using a micro-hematocrit method (Hettich 210, DJB Labcare, Newport Pagnell, UK). The remaining blood was placed in test tubes to separate the plasma (BD Vacutainer PPT™ Plasma Preparation Tube, Oxford, UK) and serum (BD Vacutainer™ Serum Tube, Oxford, UK). The plasma was obtained by centrifuging the tubes for 10 min at 1000× *g* and 4 °C (SIGMA 2-16KL, Sigma Laborzentrifugen GmbH, Osterode am Harz, Germany). The erythrocyte sediments obtained during this process were washed three times with cold saline (4 °C). To extract the serum, the test tubes were allowed to stand for 30 min for the blood to clot and then centrifuged at 1000× *g* and 4 °C. The blood plasma, serum, and erythrocytes were stored at −80 °C for less than one month before they were assayed. The activity of the following antioxidant enzymes (superoxide dismutase—SOD, EC 1.15.1.1; glutathione peroxidase—GPx, EC 1.11.1.9; catalase—CAT, EC 1.11.1.6) was analyzed. The SOD activity was measured using a commercially available RANSOD SD125 kit (Randox, Crumlin, UK), and the intra- and inter-assay CVs for SOD were 4.11% and 6.51%, respectively. Meanwhile, the activity of GPx was assayed using a commercially available RANSEL RS505 kit (Randox, UK), and the intra- and inter-assay CVs for GPx were 5.83% and 4.03%, respectively. The activity of CAT was assayed using the Aebi method [29].

The activities of creatine kinase (CK, 2.7.3.2) and lactate dehydrogenase (LDH, EC 1.1.1.27) and the concentration of uric acid (UA) in the plasma samples were determined using Randox Laboratories diagnostic kits (Randox, Crumlin, UK; CK522, LD401 and UA230, respectively). The concentration of UA was determined using a colorimetric method. The intra- and inter-assay coefficients of variation (CVs) were 1.93% and 3.63% (CK), 2.83% and 3.38% (LDH), and 0.38% and 5.64% (UA). The plasma lipid peroxide concentrations were assayed using the thiobarbituric acid (TBA) test following Buege and Aust’s method [30], which we modified by adding 0.01% butylated hydroxytoluene to lower the metal-catalyzed auto-oxidation of lipids during heating with the TBA reagent. The chromogen was extracted using n-butanol, and the absorbance of the organic layer was read at 532 nm [12]. The lipid peroxide concentrations were expressed as micromoles of malondialdehyde (MDA) per liter of plasma, calculated from a calibration curve prepared using 1,1,3,3-tetraethoxypropane. The serum total antioxidant capacity (TAS) was determined using a colorimetric method (NX2332, Randox, UK). The intra- and inter-assay CVs for the TAS were 1.20% and 1.77%, respectively. The total oxidative capacity (TOS) in the plasma was determined using an immunoenzymatic method employing a diagnostic kit (Immundiagnostic PERox (TOS/TOC), Bensheim, Germany, cat. no. KC5100). The serum cortisol concentration was determined at a central external diagnostic laboratory, using an immunochemical method employing the Abbott Alinity ci-series (08P33, Abbott, Chicago, IL, USA).

All the biochemical tests were conducted in accordance with PN-ENISO 9001:2015 (certificate no. PW19912-18B) and the manufacturers’ instructions by a certified biochemistry laboratory.

### 2.3. Statistical Analysis

The data are presented as the means and standard deviations (Ms ± SDs). The normality of the differences between paired data points was assessed using the Shapiro–Wilk test (*W*). To compare results before and after surgery, the paired-samples *t* test and the Wilcoxon signed-rank test were applied, depending on data distribution. Holm’s *p*-value correction was applied to control for multiple testing and the family-wise error rate. Effect sizes were calculated using Cohen’s *d* with Dunlap’s correction (*d_c_*) and Rosenthal’s correlation coefficient (*r*). We interpreted the effect size based on the *d_c_* value as follows: 0.20–<0.50 indicated a small effect, 0.50–<0.80 indicated a medium effect, and ≥0.80 indicated a large effect. For *r* values, <0.20 indicated a very small effect, 0.20–<0.40 indicated a small effect, 0.40–<0.60 indicated a moderate effect, 0.60–<0.80 indicated a strong effect, and ≥0.80 indicated a very large effect. The significance level for all statistical tests was set at α = 0.05. Statistical analyses were conducted using IBM SPSS Statistics (Version 26.0; IBM Corp., Armonk, NY, USA).

## 3. Results

A paired-samples *t* test and the Wilcoxon signed-rank test were conducted to determine whether patients’ red blood cell indices and cortisol blood concentrations after surgery differed from those before surgery. The results showed no significant (*p* > 0.05) changes in hemoglobin (Hb), hematocrit (Ht) and cortisol (Cor) (Table 2).

### 3.1. Blood Prooxidant–Antioxidant Balance

The results of the antioxidant status (SOD, CAT, GPx, GSH, UA, TAS) and oxidative stress indicators (MDA, TOS) are summarized in Table 3. A paired-samples *t* test and the Wilcoxon signed-rank test were conducted to determine whether patients’ blood prooxidant–antioxidant balance after surgery differed from that before surgery. The activities of SOD, CAT, and GPx in patients after surgery were not significantly different (*p* > 0.05) from those recorded before surgery. The GSH concentrations were significantly lower (*t* = 3.07, *p* = 0.038, *d_c_* = 0.78) after the surgery. The plasma concentrations of MDA following surgery were significantly lower (*Z* = −3.58, *p* = 0.001, *r* = 0.80) than those measured after the surgery. Serum TOS levels were significantly elevated in all patients post-surgery compared to pre-surgery (*t* = −2.15, *p* = 0.044, *d_c_* = 0.41) (Table 3).

The normality of differences between pairs 1 and 2 was as follows: SOD, W = 0.94, *p* = 0.198; CAT, W = 0.95, *p* = 0.330; GPx, W = 0.96, *p* = 0.548; GSH, W = 0.95, *p* = 0.415; TAS, W = 0.91, *p* = 0.062; UA, W = 0.97, *p* = 0.846; MDA, W = 0.89, *p* = 0.033; TOS, W = 0.97, *p* = 0.715.

### 3.2. Activity of CK and LDH

A paired-samples *t* test showed that no significant changes (*p* > 0.05) were observed in the leakage of intracellular enzymes (CK, LDH) into the patients’ circulatory systems (Table 4).

## 4. Discussion

The purpose of this study was to determine the effect of surgery on the blood prooxidant–antioxidant balance, red blood cell indicators, and cortisol concentrations in patients with benign parotid tumors, as well as the levels of cellular enzymes measurable in their blood plasma. Antioxidant mechanisms, when their functions are impaired, can lead to chronic inflammation, which in turn can contribute to carcinogenesis [31].

It has been very well documented in the literature that chronic inflammation can have a significant impact on tumor development, from tumor initiation and promotion to progression and metastasis [2,32], and inflammatory cytokines and chemokines play a key role in modulating the immune microenvironment in parotid tumors [2]. These changes can affect tumor progression, immune cell infiltration, and their usefulness as diagnostic indicators [2,4,33]. Chemokines are released from resident immune cells at the site of injury and stimulate a gradient that induces leukocyte migration [33]. In patients with salivary gland tumors (WT and PA), various chemokines have been determined, including CCL28 [34], CXCL10, CXCL12, CCL18 [35], and CXCL1 [36], as well as pro-inflammatory cytokines such as IL-1β [36], IL-6 [25], IL-4 [37], IL-17 [38], and IL-33 [25,39,40,41]. Based on current data, IL-33 may have potential as a biomarker for the diagnosis of salivary gland tumors [2].

The surgical treatment of parotid gland tumors includes various types of parotidectomy. Surgical tissue damage induces an inflammatory response, thus leading to the release of pro-inflammatory molecules. It increases the production of ROS and reduces the function of antioxidant defense mechanisms, leading to postoperative oxidative stress [42]. Moreover, antioxidant enzymes and molecules are potential treatment targets in malignant diseases [43]. Only a small number of studies have examined the efficacy of controlling the antioxidant and oxidative status in salivary gland tumor patients [4].

The most commonly used indicators of oxidative stress include elevated levels of oxidation products derived from lipids, proteins, and nucleic acids, along with reduced concentrations of antioxidants [44]. The assessment of oxidative stress, along with the enhancement of the antioxidant defense system, may be of significant importance in the prevention and treatment of carcinogenesis [45]. The relationship between oxidative stress and cancer is still incompletely understood and is considered a multifactorial phenomenon. However, many oncologic pathologies have shown well-established correlations with elevated levels of oxidative stress markers [4]. Only a small number of studies have examined the efficacy of controlling antioxidant and oxidative status in salivary gland tumor patients [4]. Antioxidant enzymes have been linked to the mechanisms responsible for maintaining prooxidant–antioxidant balance. Sowa et al. (2018) [4] evaluated the activity of SOD and its isoenzymes, i.e., Mn-SOD and Cu/Zn-SOD, in patients with WA and PA. In the WT group, Sowa et al. (2018) [4] observed a decrease in the antioxidant enzyme Cu/Zn-SOD. It suggested the involvement of this antioxidant enzyme in the pathogenesis of this type of tumor. Based on these results, it can be speculated that a decrease in Mn-SOD is associated with carcinogenesis and SOD activity may play a key role in radiation therapy [4,46].

Indicators of oxidative damage to proteins were evaluated in patients with parotid gland tumors. The most commonly analyzed were the levels of thiol groups, whose reduction indicates increased oxidative stress. Reduced levels of low-molecular-weight thiols were found in the serum of patients, with protein thiols being the predominant fraction [4,47]. According to Sowa et al. (2018) [4], low levels of systemic oxidative stress were observed in patients with benign salivary gland tumors. This is confirmed by reduced values of total antioxidant capacity by the FRAP method and reduced concentrations of thiol groups, as well as elevated levels of advanced protein oxidation products (AOPPs) [2]. In addition, AOPPs prove to be a useful marker of oxidative protein damage in patients with parotid gland tumors [4].

Carcinoma ex pleomorphic adenoma (CXPA) is a rare malignant transformation observed in the parotid gland. The etiology of CXPA remains poorly understood, though alterations in the expression of the PLAG1 gene have been implicated as a potential contributing factor [7]. However, there is currently a lack of scientific evidence supporting the use of oxidative stress markers in patients with giant parotid tumors.

In our study, surgical intervention did not lead to a statistically significant enhancement of enzymatic antioxidant defense mechanisms assessed on day 10 after surgery. Postoperative measurements showed only marginal, non-significant increases in antioxidant enzymes such as SOD, CAT and GPx. Comparable findings were reported by Misiak et al. (2014) [31], who noted elevated levels of SOD, CAT, and GPx following radical lung cancer surgery. Similarly, Kärkkäinen et al. (2018) [48] documented a significant rise in plasma SOD concentrations within the first 24 h after abdominal procedures involving laparotomy. Assessment of GSH levels in patients with salivary gland tumors revealed a significant postoperative decrease. Tumor development, including that of salivary gland neoplasms, is frequently associated with the excessive generation of ROS. GSH, the principal non-enzymatic intracellular antioxidant, plays a critical role in neutralizing ROS. Under conditions of sustained oxidative stress, indicated by elevated TOS, GSH reserves may become progressively depleted, reflecting increased antioxidant demand and impaired redox homeostasis [49].

Numerous studies have demonstrated a significant postoperative increase in serum MDA levels in patients undergoing cardiac, orthopedic, and ophthalmic surgeries [42,50]. In contrast, research on perioperative MDA dynamics in patients with parotid gland tumors is limited and yields inconclusive results.

Sowa et al. (2018) [4] reported a trend toward elevated MDA levels in patients with WT and PA compared to healthy controls. In a different context, Didžiapetrienė et al. (2020) [51] found a significant reduction in the MDA levels five days after breast cancer surgery. Similarly, Bozan et al. (2018) [52] observed significantly increased the MDA concentrations in patients with laryngeal cancer, followed by a marked decline after laryngectomy. These findings support the hypothesis that MDA may play a role as a potential mediator of carcinogenesis [52,53,54].

In our study, we observed a statistically significant decrease in the plasma MDA levels by postoperative day 10 in the patients with benign parotid gland tumors, contrasting with the trends reported in other surgical contexts.

Findings on surgery-induced oxidative stress markers remain inconclusive and appear to vary based on both the surgical technique employed and the timing of blood sample collection for biochemical analysis. For instance, Arsalani-Zadeh et al. (2011) [55] reported significant differences in oxidative stress markers when comparing patients undergoing laparoscopic versus open abdominal surgery.

The lack of significant changes in the blood TAS observed 10 days postoperatively was accompanied by a substantial increase in the TOS. These results align with the findings of Koksal and Kurban (2010) [56], who reported a comparable, non-significant elevation in TAS one day after laparoscopic cholecystectomy. The observed increase in oxidative stress may be attributable to sustained physiological stress associated with the surgical intervention. Although the body attempts to upregulate antioxidant mechanisms in response to oxidative stress, this response may be inadequate or delayed. Furthermore, chronic ROS exposure may lead to depletion of endogenous antioxidants (e.g., glutathione), resulting in minimal observable change in TAS [57].

No statistically significant changes in CK and LDH activities were observed in the patients following surgery. To date, there is a notable lack of data in the literature specifically on CK and LDH activities in individuals with parotid gland tumors. However, existing evidence from other malignancies suggests a potential role for these enzymes in tumor progression and prognosis. Liu et al. (2017) [58] demonstrated that the plasma levels of CK and LDH are closely associated with disease advancement in breast, prostate, and colorectal cancers. LDH, in particular, is widely recognized as a biomarker of tissue damage resulting from tumor growth, with both serum and plasma concentrations being of diagnostic relevance [59]. Furthermore, Al-Daami et al. (2021) [60] suggested that elevated CK and LDH activity in cancer patients may serve as a negative prognostic indicator, potentially correlating with reduced overall survival. Surgery is a stress factor with varying impacts according to its duration and induces a controlled systemic stress response that includes a wide range of endocrine, immune and cardiovascular effects, including increased cortisol production in the adrenal glands [61]. Although no statistically significant changes in the patients’ cortisol levels were found, a slight (non-significant) decrease (−19%) was recorded. The findings from a study by Kwon et al. (2019) [62] demonstrated that the cortisol levels rose sharply in patients following hip surgery and subsequently declined gradually over time. Our result may indicate a certain tendency for cortisol levels to decrease for a few or more days after surgery. The cortisol response to postoperative stress varies significantly in patients depending on the invasiveness of the procedure [61].

The hemoglobin level is an important determinant of the prognosis in patients with parotid gland tumors [63]. In the case of head and neck cancers, research has been conducted on the most common squamous cell carcinomas, particularly laryngeal cancer [64]. A slight (non-significant) increase in Hb (+5%) and Ht (+4%) was recorded 10 days after surgery. A similar result was obtained by Chen et al. (2015) [65], who observed an increase in the hemoglobin and hematocrit levels in patients 10 days after a total hip and knee arthroplasty.

## 5. Conclusions

The surgical removal of salivary gland tumors did not result in a reduction in oxidative stress on postoperative day 10. Despite a pronounced decrease in MDA concentrations, a significant increase in the TOS was observed in the patients following salivary gland excision. This paradoxical increase in oxidative stress may be attributed to a concomitant and significant decline in GSH levels in patients undergoing tumor resection. Nevertheless, further studies are warranted to elucidate the underlying mechanisms and validate these observations, as well as to determine the effectiveness of endogenous defense mechanisms in counteracting oxidative stress induced by salivary gland tumors.

## 6. Study Limitations

This study has several limitations. First, the analysis did not include an assessment of prooxidant–antioxidant balance markers in parotid tumor tissue homogenates, and the current protocol, with blood collection only on day 10, omits the critical early postoperative window (2–24 h), during which oxidative stress markers peak. Second, the absence of a control group limited the study to an observational design. Therefore, future research should consider incorporating randomized controlled trial (RCT) methodologies to enhance causal inference. Lastly, increasing the sample size in subsequent studies would improve statistical power and the overall reliability of the findings.

## Figures and Tables

**Table 1:** Basic anthropometric characteristics of patients.

Variable	M ± SD
Age (years)	63.9 ± 12.4
Body weight (kg)	79.9 ± 18.5
Body height (cm)	168.6 ± 7.67
BMI (kg/m^2^)	28.1 ± 5.98

Notes: M—mean; SD—standard deviation; BMI—body mass index.

**Table 2:** Changes in Hb, Ht and Cor levels after surgery.

Variables	Time	M ± SD	*t*	*p*	*d_c_*
Hb (g/dL)	1	14.2 ± 1.7	−1.67	0.112	0.48
	2	14.9 ± 1.0			
Ht (%)	1	40.5 ± 5.1	−1.18	0.252	0.36
	2	42.0 ± 2.7			
Cor (µg/dL)	1	13.4 ± 4.1	2.05	0.054	0.64
	2	10.9 ± 3.5			

Notes: M, mean; SD, standard deviations; 1, before surgery; 2, after surgery; *t*, paired-samples *t* test; *p*, adjusted probability value for Z test; *dc*, effect size for paired-samples *t* test (Cohen’s d with Dunlap correction); Hb, hemoglobin; Ht, hematocrit; Cor, cortisol. The normality of differences between pairs 1 and 2: Hb, W = 0.74, *p* < 0.001; Ht, W = 0.69, *p* < 0.001; Cor, W = 0.92, *p* = 0.112.

**Table 3:** Blood prooxidant–antioxidant balance after surgery.

Variables	Time	M ± SD	Statistics	Significant	Effect Size
SOD (U/gHb)	1	1414.1 ± 254.4	*t* = −0.31	*p* = 1.00	*d_c_* = 0.09
	2	1436.3 ± 212.7			
CAT (U/gHb)	1	167.0 ± 29.4	*t* = −0.22	*p* = 0.100	*d_c_* = 0.06
	2	168.9 ± 30.5			
GPx (U/gHb)	1	39.9 ± 8.4	*t* = −0.11	*p* = 0.916	*d_c_* = 0.04
	2	40.2 ± 8.7			
GSH (µg/mgHb)	1	3.0 ± 0.4	*t* = 3.07	*p* = 0.038	*d_c_* = 0.78
	2	2.7 ± 0.3			
TAS (mmol/L)	1	1.1 ± 0.3	*t* = −1.54	*p* = 0.563	*d_c_* = 0.48
	2	1.2 ± 0.2			
UA (mg/dL)	1	5.8 ± 1.5	*t* = 2.03	*p* = 0.281	*d_c_* = 0.29
	2	5.3 ± 1.5			
MDA (µmol/L)	1	6.2 ± 1.4	*Z* = −3.58	*p =* 0.001	*r* = 0.80
	2	4.3 ± 1.1			
TOS (µmol/L)	1	430.0 ± 208.7	*t* = −2.15	*p* = 0.044	*d_c_* = 0.41
	2	520.1 ± 229.6			

Notes: M, mean; SD, standard deviations; 1, before surgery; 2, after surgery; *t*, paired-samples *t* test; Z, Wilcoxon signed-rank test (standardized test statistic); *p*, adjusted probability value for *t* test/Z test; *dc*, effect size for paired-samples *t* test (Cohen’s d with Dunlap correction); *r*, effect size for Wilcoxon signed-rank test (Rosenthal correlation coefficient). SOD—superoxide dismutase; CAT—catalase; GPx—glutathione peroxidase; GSH—reduced glutathione; TAS—total antioxidant capacity; UA—uric acid; MDA—malondialdehyde; TOS—total oxidative capacity.

**Table 4:** Changes in cytoplasmic enzymes detected in the bloodstream.

Variables	Time	M ± SD	Statistics	Significant	Effect Size
CK (U/L)	1	120.3 ± 99.5	*t* = 0.90	*p* = 0.760	*d_c_* = 0.23
	2	100.0 ± 75.5			
LDH (U/L)	1	797.9 ± 335.2	*t* = 0.69	*p* = 0.500	*d_c_* = 0.22
	2	726.4 ± 306.6			

Notes: M, mean; SD, standard deviations; 1, before surgery; 2, after surgery; *t*, paired-samples *t* test; *p*, adjusted probability value for *t* test; *dc*, effect size for paired-samples *t* test (Cohen’s d with Dunlap correction); CK—creatine kinase; LDH—lactate dehydrogenase. The normality of differences between pairs 1 and 2: CK, W = 0.93, *p* = 0.186; LDH, W = 0.98, *p* = 0.893.

## Data Availability

The raw data supporting the conclusions of this article will be made available by the authors on request.

## Abbreviations

The following abbreviations are used in this manuscript:

ANZCTR	Australian New Zealand Clinical Trials Registry
AOPPs	Advanced protein oxidation products
BMI	Body mass index
CAT	Catalase
CK	Creatine kinase
Cor	Cortisol
CT	Computed tomography
CV	Coefficients of variation
CXPA	Carcinoma ex pleomorphic adenoma
GPX	Glutathione peroxidase
GSH	Reduced glutathione
Hb	Hemoglobin
HPV	Human papillomavirus
Ht	Hematocrit
LDH	Lactate dehydrogenase
MDA	Malondialdehyde
PA	Pleomorphic adenoma
ROS	Reactive oxygen species
SOD	Superoxide dismutase
TAS	Total antioxidant capacity
TBA	The thiobarbituric acid
TOS	Total oxidative capacity
UA	Uric acid
WT	Warthin’s tumor
